# A Rapid Method for Simultaneous Screening of Multi-Gene Mutations Associated with Hearing Loss in the Korean Population

**DOI:** 10.1371/journal.pone.0057237

**Published:** 2013-03-01

**Authors:** Borum Sagong, Jeong-In Baek, Se-Kyung Oh, Kyung Jin Na, Jae Woong Bae, Soo Young Choi, Ji Yun Jeong, Jae Young Choi, Sang-Heun Lee, Kyu-Yup Lee, Un-Kyung Kim

**Affiliations:** 1 Department of Biology, College of Natural Sciences, Kyungpook National University, Daegu, South Korea; 2 Department of Otorhinolaryngology-Head and Neck Surgery, College of Medicine, Kyungpook National University, Daegu, South Korea; 3 Department of Medicine, University of Pennsylvania, Philadelphia, Pennsylvania, United States of America; 4 Department of Endocrinology and Metabolism, Kyungpook National University Hospital, Kyungpook National University School of Medicine, Daegu, South Korea; 5 Department of Otorhinolaryngology, Yonsei University College of Medicine, Seoul, South Korea; University Hospital Vall d'Hebron, Spain

## Abstract

Hearing loss (HL) is a congenital disease with a high prevalence, and patients with hearing loss need early diagnosis for treatment and prevention. The *GJB2*, *MT-RNR1*, and *SLC26A4* genes have been reported as common causative genes of hearing loss in the Korean population and some mutations of these genes are the most common mutations associated with hearing loss. Accordingly, we developed a method for the simultaneous detection of seven mutations (c.235delC of *GJB2*, c.439A>G, c.919-2A>G, c.1149+3A>G, c.1229C>T, c.2168A>G of *SLC26A4*, and m.1555A>G of the *MT-RNR1* gene) using multiplex SNaPshot minisequencing to enable rapid diagnosis of hereditary hearing loss. This method was confirmed in patients with hearing loss and used for genetic diagnosis of controls with normal hearing and neonates. We found that 4.06% of individuals with normal hearing and 4.32% of neonates were heterozygous carriers. In addition, we detected that an individual is heterozygous for two different mutations of *GJB2* and *SLC26A4* gene, respectively and one normal hearing showing the heteroplasmy of m.1555A>G. These genotypes corresponded to those determined by direct sequencing. Overall, we successfully developed a robust and cost-effective diagnosis method that detects common causative mutations of hearing loss in the Korean population. This method will be possible to detect up to 40% causative mutations associated with prelingual HL in the Korean population and serve as a useful genetic technique for diagnosis of hearing loss for patients, carriers, neonates, and fetuses.

## Introduction

Congenital hearing loss (HL) is a common sensory impairment that occurs in approximately one in 1000 neonates, and more than 50% of cases are hereditary [1]. The occurrence of HL in early childhood could have serious effects on language acquisition, while later onset of severe hearing defect compromises sociality [2]. Currently, the best provision for hereditary HL is prevention of outbreak through early diagnosis and continuous management. Therefore, a precise rapid genetic diagnosis may be the most effective support for treatment and prevention of HL.

More than over 150 loci have been mapped in HL, and 57 genes of them have been shown to cause nonsyndromic HL [3]. Among these, mutations of the *GJB2* gene (OMIM 121011) are the most common cause of hereditary HL in many Caucasian populations [4]. One of them, mutation c.35delG, accounts for up to 70% of the pathologic alleles in European and Caucasian populations [5]. Unlike Caucasian populations, *GJB2* mutations seem to account for a lower percentage of hereditary HL in East Asian populations; however, approximately 43% of the *GJB2* hearing loss are caused by c.235delC mutation in Koreans [6]–[8]. Moreover, mutations of the *SLC26A4* gene (OMIM 605646), which cause both Pendred syndrome and nonsyndromic HL with enlarged vestibular aqueduct, appear to be common in Asian populations [9]. Especially, five mutations of this gene account for up to 90% of all mutant alleles [8], [10]. Mitochondrial DNA mutations have also been reported in both nonsyndromic and syndromic HL. Among these mutations, only the m.1555A>G mutation of the *MT-RNR1* gene (OMIM 561000) has been identified repeatedly in Koreans with HL by our previous studies [11], [12]. Based on the prevalence of mutations from the previous studies, we selected 7 mutations which show the high frequencies in Korean hearing loss population and designed the method to detect effectively mutant alleles through rapid screening: c.235delC of *GJB2*, c.919-2A>G and c.2168A>G of *SLC26A4* and m.1555A>G of the *MT-RNR1* gene. All of the 7 mutations selected in this study had been reported that mutations cause abnormal function. The c.235delC mutation of *GJB2* gene caused a loss of targeting activity to the cell membrane and severe deterioration of gap junction activity [13]. The m.1555A>G in *MT-RNR1* gene made the human mitochondrial ribosome more bacteria-like and alter binding sites for aminoglycosides [14]. The p.M147V and p.H723R mutations of *SLC26A4* gene caused loss of Cl^−^/HCO_3_^−^ and Cl^−^/I^−^ exchange activity [15]–[17]. The p.T410M mutation in *SLC26A4* gene caused loss of iodide efflux [17], [18]. The c.919-2A>G and c.1149+3A>G mutations of *SLC26A4* gene resulted in premature termination of translation on gene expression, respectively [10], [19]. Accordingly, these mutations have been screened by priority during genetic testing of HL. Because all of these mutations are recessive in their effect except the mitochondrial mutation (m.1555A>G), analysis of individual genotype for these mutations can provide crucial genetic information for inheritance of autosomal recessive hearing loss.

In this study, we developed a rapid and low cost multiplex genetic diagnosis of common mutations in Koreans using SNaPshot minisequencing for patients with HL and heterozygous carriers.

## Materials and Methods

### Subjects and DNA preparation

To set up the SNaPshot minisequencing methods, five patients with mutations in *GJB2*, *MT-RNR1*, and *SLC26A4* and one normal hearing individual without any mutations were used as positive and normal controls, respectively. One hundred ninety-seven unrelated Koreans with normal hearing from Kyungpook National University Hospital were used as controls aged 20 and 64 years. In addition, 139 Korean neonates of unrelated individuals who were born in Kyungpook National University Hospital were recruited for this study with the consent of their parents. Written informed consent was obtained from all participants, and this study was approved by the local ethics committee.

The genomic DNA of normal controls and neonates was extracted from peripheral blood and buccal cells using a FlexiGene DNA kit (Qiagen, Hilden, Germany) or a Gentra Puregene Buccal Cell kit (Qiagen, Hilden, Germany) following the manufacturer's instructions.

### PCR multiplex amplification

Fourteen primers were used for amplification of the mutation region, and amplification was conducted in a reaction mixture with a final volume of 25 μl that contained 20 ng of DNA template, 0.2 mM deoxynucleotide, 1 X multiplex polymerase chain reaction (PCR) primer mixture that consisted of seven pairs of primers, 1 X h-Taq reaction buffer, and 0.5 U of h-Taq DNA Polymerase (Solgent, Daejeon, Korea) (Table 1). The PCR conditions were as follows: initial denaturation at 95°C for 15 min, followed by 40 cycles of denaturation at 94°C for 20 sec, annealing at 57°C for 20 sec, and extension at 72°C for 40 sec and then final extension at 72°C for 5 min. Five microliters of the PCR products were separated and visualized following electrophoresis on a 2% agarose gel. Shrimp alkaline phosphatase (SAP) (USB, Cleveland, OH, USA) and exonuclease I (USB, Cleveland, OH, USA) were used for post-PCR purification of the examined PCR products.

**Table 1:** Multiplex PCR primer sequences.

Gene	Exon	PCR primers (5′→3′)		Concentration* (pmol/μl)	Product size (bp)	Nucleotide change	Protein change
*GJB2*	2	F	TCTTTTCCAGAGCAAACCGC	0.2	416	c.235delC	p.L79CfsX3
		R	GATGCGGACCTTCTGGGTTT	0.2			
*MT-RNR1*	-	F	CGTCACCCTCCTCAAGTATACTTC	0.04	137	m.1555A>G	-
		R	GCTTTGTGTTAAGCTACACTCTGG	0.04			
*SLC26A4*	5	F	TTTTTAAACCCTATGCAGACACA	0.5	175	c.439A>G	p.M147V
		R	TTAATACAGTTCCATTGCTGCTG	0.5			
*SLC26A4*	7	F	CAAAATCCCAGTCCCTATTCCTA	0.4	363	c.919-2A>G	-
		R	GGTTGTTTCTTCCAGATCACACAC	0.4			
*SLC26A4*	9	F	GCTTGTTCTCGGAGATGCTG	0.15	301	c.1149+3A>G	-
		R	AGTGATGCAGTGTGTCTATTCC	0.15			
*SLC26A4*	10	F	GGATCGTTGTCATCCAGTCTC	0.5	488	c.1229C>T	p.T410M
		R	TTACCAGGCCATCTGTCTCC	0.5			
*SLC26A4*	19	F	CCTGGGCAATAGAATGAGACTC	0.15	227	c.2168A>G	p.H723R
		R	AAATGGAACCTTGACCCTCTTG	0.15			

* Final concentration in the reaction mixture.

### SNaPshot minisequencing reaction

The single base extension (SBE) reaction was performed in a reaction mixture with a final volume of 15 μl that contained 2.45 μl of purified multiplex PCR product, 1 X extension primer mixture (Table 2), and 3 μl of SNaPshot Multiplex Ready Reaction Mix (Applied Biosystems, Foster City, CA, USA). The reaction mixture was subjected to 30 SBE cycles of denaturation at 96°C for 10 sec, primer annealing at 50°C for 5 sec, and primer extension at 60°C for 30 sec. SAP (USB, Cleveland, OH, USA) was used for post-SBE purification of the SBE reaction products.

**Table 2:** Extension primer sequences.

Gene	Mutation	Extension primers (5′→3′)		Concentration* (pmol/μl)	Primer size (bp)	WT allele (peak color)	MT allele (peak color)
*GJB2*	c.235delC	F	ACGATCACTACTTCCCCATCTCCCACATCCGGCTATGGGCC	4	41	C (Black)	T (Red)
*MT-RNR1*	m.1555A>G	F	TTAACTAAAACCCCTACGCATTTATATAGAGGAG	0.07	34	A (Green)	G (Blue)
*SLC26A4*	c.439A>G	F	TTAATAACTGATTAATTGTTAGAGACTTTTTTTCCCCAGGA CCTTTTCCAGTGGTGAGTTTA	0.05	62	A (Green)	G (Blue)
*SLC26A4*	c.919-2A>G	F	AAGTTCAGCATTATTTGGTTGACAAACAAGGAATTATTAAA ACCAATGGAGTTTTTAACATCTTTTGTTTTATTTC	0.03	76	A (Green)	G (Blue)
*SLC26A4*	c.1149+3A>G	R	TATGTTTTTTTCCTGTTTCCAGCCCTATAAAACCAGTTCAGCA AAAGGGCACCCA	0.07	55	A (Red)	G (Black)
*SLC26A4*	c.1229C>T	F	CCTTTGGGATCAGCAACATCTTCTCAGGATTCTTCTCTTGTTTT GTGGCCACCACTGCTCTTTCCCGCA	6.67	69	C (Black)	T (Red)
*SLC26A4*	c.2168A>G	F	GGTTCTTTGACGACAACATTAGAAAGGACACATTCTTTTTGACG GTCC	0.07	48	A (Green)	G (Blue)

* Final concentration in the reaction mixture.

For electrophoresis, 2 μl of purified multiplex SBE reaction products were mixed with 0.3 μl of GeneScan 120 LIZ Size Standard (Applied Biosystems, Foster City, CA, USA) and 7.7 μl of Hi-Di Formamide (Applied Biosystems, Foster City, CA, USA) and denatured at 95°C for 2 min. The fluorescently labeled fragments were resolved by capillary electrophoresis on an ABI 3130*xl* Genetic Analyzer (Applied Biosystems, Foster City, CA, USA). The resulting data were analyzed with the GeneMapper v3.7 (Applied Biosystems, Foster City, CA, USA) software.

### Sanger sequencing

Specific DNA fragments containing each mutation were amplified by PCR to confirm the genotype. PCR was conducted using a reaction mixture composed of 0.2 mM deoxynucleotide, 10 pmol of each forward and reverse primer, and 0.25 U of h-Taq DNA Polymerase (Solgent, Daejeon, Korea) in a final volume of 25 μl. The PCR conditions were as follows: initial denaturation at 95°C for 15 min, followed by 35 cycles of denaturation at 94°C for 20 sec, annealing at 55°C, depending on the primers for 40 sec, and extension at 72°C for 50 sec followed by final extension at 72°C for 5 min. The quality of the PCR products was examined by electrophoresis on 2% agarose gels. SAP (USB, Cleveland, OH, USA) and exonuclease I (USB, Cleveland, OH, USA) were used for purification of the examined PCR products. The sequences of the purified PCR products were obtained by direct sequencing using a BigDye Terminator v3.1 Cycle Sequencing Kit (Applied Biosystems, Foster City, CA, USA). Extended products were purified by ethanol precipitation. A 3130*xl* Genetic Analyzer (Applied Biosystems, Foster City, CA, USA) was used to resolve the products and the data were analyzed using the Chromas Lite v2.01 (Technelysium Pty Ltd., Tewantin, QLD, Australia) software.

## Results

### Development of the multiplex amplification and multiplex SNaPshot minisequencing

**Figure 1:**
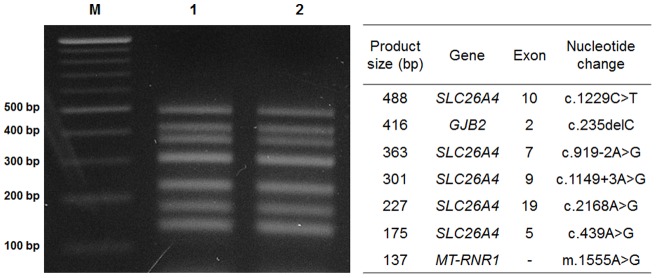
The products of multiplex amplification. Agarose gel electrophoresis pattern of digested PCR products stained with ethidium bromide shows the multiplex amplification products of wild type (lane 1), mutant type (lane 2), 100 bp ladder marker (lane M), and corresponding size (bp) for each band of multiplex PCR products (table).

**Figure 2:**
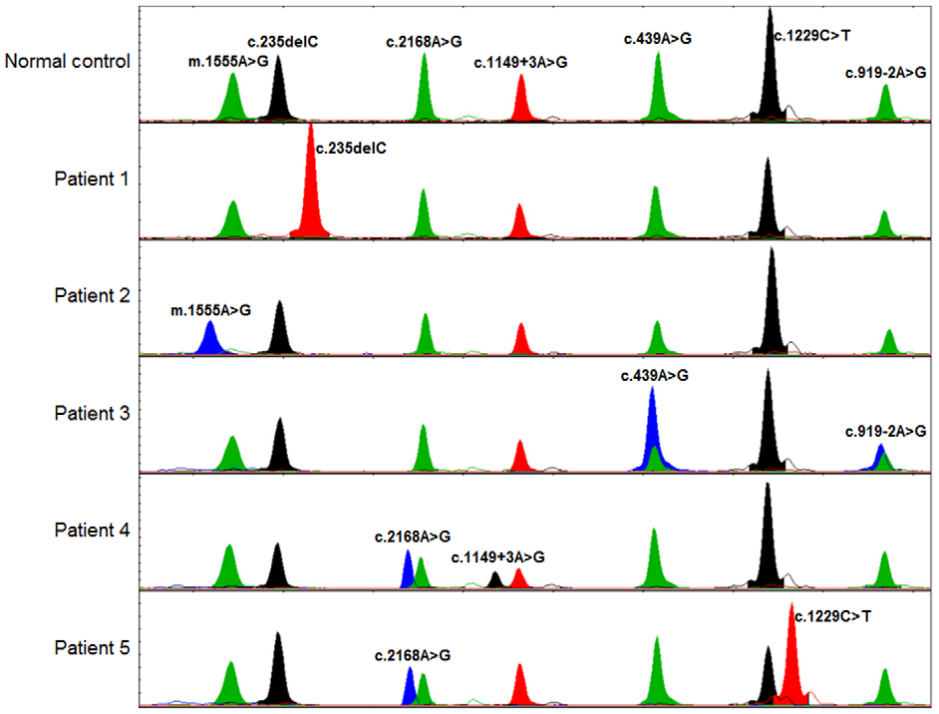
Electropherograms of Genescan Analysis of the SNaPshot multiplex minisequencing. On the top, a control DNA sample with the position and nucleotide of the wild type allele indicated. Following, electropherograms for the most frequent hearing loss mutations in homozygote, compound heterozygote forms on *GJB2*, *SLC26A4* genes, and homoplasmy in the *MT-RNR1* gene.

The seven genomic segments containing each mutation were amplified in a single multiplex PCR reaction, after which the amplified DNA fragments were separated and visualized on agarose gel (Figure 1). We developed a genetic diagnostic technique consisting of SNaPshot multiplex minisequencing of seven mutations in three genes. Five positive controls for the mutant allele of the seven mutations and one normal control with wild-type alleles were analyzed and correctly genotyped in all reactions (Figure 2). Normal controls were genotyped to wild-type in every mutant region (Figure 2, first panel). Positive controls were genotyped mutant types in each mutant region (Figure 2, second to sixth panels). Relative differences in peak heights between each allele were observed because fluorescence emission can be directly influenced by interaction between other fluorophores. In addition, the results of SNaPshot minisequencing were compared with those of Sanger sequencing of control samples, and these results were matched exactly.

### SNaPshot minisequencing in the normal hearing controls and neonates

To investigate the frequency of these common mutations in the Korean normal hearing population, the genotypes of 197 normal hearing controls and 139 neonates were analyzed for the seven mutations using SNaPshot multiplex minisequencing. Analysis revealed a 20-year-old female with normal hearing that had heteroplasmy of m.1555A>G in the *MT-RNR1* gene, which is the most common cause of aminoglycoside-induced HL. In addition, a 35-year-old female with normal hearing was found to have two different heterozygous alleles (c.235delC in *GJB2* and c.2168A>G in *SLC26A4* gene). Moreover, eight normal hearing controls and six neonates were diagnosed as carriers of each mutation. In the normal hearing controls, c.235delC of the *GJB2* gene and c.919-2A>G and c.2168A>G of the *SLC26A4* gene mutations were detected in one, two and four heterozygotes, respectively (Table 3, left panel). In the neonates, three, two and one individuals were found to be heterozygous carriers of the c.235delC *GJB2* gene mutation, c.919-2A>G and c.2168A>G *SLC26A4* mutation, respectively (Table 3, right panel). These genotypes diagnosed by the SNaPshot multiplex minisequencing matched those determined by direct sequencing.

**Table 3:** Frequencies of seven hearing loss mutations in normal controls and neonates.

Gene	Mutation	Normal hearing controls		Neonates	
		No. of alleles (n = 394)	Allele frequency (%)	No. of alleles (n = 278)	Allele frequency (%)
*GJB2*	c.235delC	2	0.51	3	1.08
*MT-RNR1*	m.1555A>G heteroplasmy	1	0.51	0	0
*SLC26A4*	c.439A>G	0	0	0	0
*SLC26A4*	c.919-2A>G	2	0.51	2	0.72
*SLC26A4*	c.1149+3A>G	0	0	0	0
*SLC26A4*	c.1229C>T	0	0	0	0
*SLC26A4*	c.2168A>G	5	1.27	1	0.36

## Discussion

The SNaPshot minisequencing technique, which was developed at first by Smith *et* *al*. [20], has been utilized in various branches of biology, including population genetics and phylogenetics, with a high level of accuracy and effectiveness. Several studies have applied this technique for haplogroup classification of human mitochondrial DNA, which provides crucial information for phylogenetic analysis and forensic identification of individuals [21], [22]. Simultaneous identification of several different species using the multiplex SNaPshot reaction has also been attempted in many studies, and their strong possibility of industrial application has been authenticated [23], [24].

Recently, SNaPshot minisequencing has been used for effective detection of numerous pathogenic mutations that cause human hereditary disorders such as *CYP21*, *PIK3CA*, *CYP3A5*, *MDR1*, and *BRCA1/2* genes [25]–[30]. Especially, it is greatly effective at genetic diagnosis of heterogeneous diseases that have a number of causative genes, as well as large single gene diseases because this technique allows simultaneous detection of several variations in a single reaction. Most heterogeneous diseases have several major common causative mutations generated from different genes. Therefore, SNaPshot multiplex minisequencing of selected common mutations provides temporal and economic efficiency rather than direct sequencing of all genes.

Based on previous genetic studies of HL in the Korean population, we selected the seven most common mutations that lead to hereditary HL [8]–[12], [31]. Multiplex genotyping of these seven mutations was successfully performed using SNaPshot minisequencing, which produced accurate data and a detection rate of the mutations that was found to account for up to 40% of the causative mutant alleles associated with prelingual HL in Koreans [8]. These results suggest that this strategy is suitable for use as a precise and rapid genetic diagnosis tool.

To verify the applicability of this technique as a genetic diagnosis tool and a tool for building a population genetic database of type and frequency of mutations in the Korean population, we analyzed genotypes of normal hearing adults and neonates. As a result, we detected heterozygous carriers successfully. Specifically, we found that 4.06% of individuals with normal hearing and 4.32% of neonates were heterozygous carriers. In addition, we identified one carrier having two different heterozygous alleles, and one normal hearing having possibility to cause the HL. The frequencies of two mutations (*GJB2* c.235delC and c.2168A>G of the *SLC26A4* gene) presented that the allele frequency of c.235delC mutation (0.52%) in normal hearing controls by other studies were very similar to the result of this study (0.51%) [10], [31]–[33], and the c.2168A>G mutation presented higher frequency than the other *SLC26A4* mutations in this study and the previous studies in Koreans [9], [10], [31]. The c.439A>G, c.1149+3A>G, and c.1229C>T mutations of *SLC26A4* were not detected in this study. Likewise, results of this study show similar results of previous studies. These findings suggest that the developed strategy can be highly effective for identification of carriers of mutations associated with HL in the Korean population. Moreover, these results indicate the potential for mitigation and prevention of HL via genetic counseling before the HL begins to possible detection of carriers having each heterozygous allele in different genes and individuals with normal hearing having genes with the potential to cause disease.

This method enables the detection of seven mutations associated with HL at once and costs less than $12 per sample from blood or buccal swab to genotype analysis (based on $1 = 1133.90 KRW), making this method rapid and cost-effective when compared with other diagnostic tools using PCR-RFLP or real-time quantitative PCR [34]–[37]. Bardien *et* *al*. elucidated that an estimated cost comparison between the two methods was less than $16 per sample for the SNaPshot method versus less than $30 per sample for the PCR-RFLP method (based on $1 = 10.39 ZAR) [37]. These results verified economical effectiveness of the SNaPshot method compared with other methods. Additionally, the analysis is quick and easy because the results of SNaPshot minisequencing show different colored peaks that reflect single bases of mutations required for analysis. Furthermore, the method can be easily performed in any location equipped with a thermal cycler and DNA sequencer. Because each mutation is simultaneously but independently amplified using specific primers, it is simple and easy to add more mutations by inserting the primers for multiplex PCR and SBE reaction. Thus, if primers are inserted for SBE reaction of other mutations identified in Korean HL, this method will be applicable to identification of genetic diagnosis of more individuals. The present science technology is very advanced, so it is possible to screen large-scale sequences in the short-term using next generation sequencing (NGS). However, the method is still somewhat expensive; therefore, known mutations should be filtered prior to NGS to identify the causative mutations. Nevertheless, our method will be fast and efficient as a method of first-pass screening. To date, this technique has been found to be useful for detection of mutations in fetal DNA from maternal plasma [38], [39], as well as both blood DNA from adults and small amounts of DNA from buccal swabs of neonates. Therefore, the developed method will make diagnosis of hereditary HL in fetuses possible as well.

Because hereditary HL is heterogeneous disorder and the major causative gene in Korean population has not been identified, it is almost impossible that all patients with genetic HL are diagnosed by single standardized diagnosis platform, even if it is highly effective techniques to detect a number of mutations. Nevertheless, three genes (*GJB2*, *SLC26A4*, and *MT-RNR1*) are the most common causative genes in Korean population, and their 7 mutations selected in this study account for up to 70% of hearing loss caused by these 3 genes [8]. It obviously suggests that detection of these 7 frequent mutations has the highest diagnosis rate than any other mutations. For this reasons, primary mutation screening of these 3 genes are essentially being required in all genetic studies of hearing loss. It means that analysis of these 7 major mutations using SNaPshot minisequencing tool is very useful for researches of hereditary hearing loss.

In conclusion, we successfully developed a rapid, accurate, robust, and cost-effective genetic tool for diagnosis of HL using SNaPshot minisequencing. It is possible to detect up to 40% causative mutations associated with prelingual HL in the Korean population using this method, and this technique is applicable to other fields including genetic diagnosis of fetuses or first-pass screening prior to NGS screening.
